# An Overview on Primary Progressive Aphasia and Its Variants

**DOI:** 10.1155/2006/260734

**Published:** 2006-07-17

**Authors:** Serena Amici, Maria Luisa Gorno-Tempini, Jennifer M. Ogar, Nina F. Dronkers, Bruce L. Miller

**Affiliations:** ^1^Memory and Aging CenterDepartment of NeurologyUniversity of CaliforniaSan FranciscoUSA; ^2^VA Northern California Health Care SystemMartinezCAUSA; ^3^University of CaliforniaDavisDavisCAUSA; ^4^Department of NeurosciencesUniversity of PerugiaPerugiaItaly

**Keywords:** Primary progressive aphasia, nonfluent progressive aphasia, semantic dementia, logopenic progressive aphasia, neuroimaging studies, language symptoms

## Abstract

We present a review of the literature on Primary Progressive Aphasia (PPA) together with the analysis of neuropschychological and neuroradiologic profiles of 42 PPA patients. Mesulam originally defined PPA as a progressive degenerative disorder characterized by isolated language impairment for at least two years. The most common variants of PPA are: (1) Progressive nonfluent aphasia (PNFA), (2) semantic dementia (SD), (3) logopenic progressive aphasia (LPA). PNFA is characterized by labored speech, agrammatism in production, and/or comprehension. In some cases the syndrome begins with isolated deficits in speech. SD patients typically present with loss of word and object meaning and surface dyslexia. LPA patients have word-finding difficulties, syntactically simple but accurate language output and impaired sentence comprehension. The neuropsychological data demonstrated that SD patients show the most characteristic pattern of impairment, while PNFA and LPA overlap within many cognitive domains. The neuroimaging analysis showed left perisylvian region involvement. A comprehensive cognitive, neuroimaging and pathological approach is necessary to identify the clinical and pathogenetic features of different PPA variants.

## 1. Introduction

Neurodegenerative dementias represent an emergent worldwide problem, brought about in part by the aging of the population. The insidious onset and the heterogeneous clinical presentation represent a diagnostic dilemma for the clinician. Dementia is characterized by progressive cognitive deficits and/or behavioral changes that impair daily-life activity (DSM-IVR, 2003). Historically, memory deficits have been considered essential for the diagnosis of dementia. Nevertheless, many dementia patients experience memory deficits only in the later stage of the disease.

An example of a non-amnestic type of dementing condition is Primary Progressive Aphasia (PPA), a slowly progressive language impairment that remains isolated for at least two years [39]. Neuroimaging typically reveals greater atrophy and/or hypometabolism within the left hemisphere compared to the right. Imaging in PPA should be negative for focal lesions such as tumor, stroke or arterio-venous malformations. At pathology, the majority of clinically diagnosed PPA patients show non-Alzheimer's disease of three major subtypes. The majority of cases show tau or ubiquitin inclusions, although a minority have no inclusions (so-called dementia lacking distinctive histopathology) [60]. The three major tau aggregation disorders include Pick's disease (PiD) [29], progressive supranuclear palsy [10,24] or corticobasal degeneration (CBD) [16,41,45]. When ubiquitin-positive inclusions are present different authors have suggested

ISSN 0953-4180/06/$17.00 © 2006 – IOS Press and the authors. All rights reserved different terminologies: frontotemporal dementia motor neuron disease (FTD-MND) if motor involvement is present; when motor involvement is not evident the various terms that have been used include frontotemporal lobar degeneration with ubiquitin-positive inclusions (FTLD-U) [48], frontotemporal lobar degeneration with inclusions that are tau and synclein-negative, ubiquitinated (FTLD with ITSNU) [30], and motor neuron disease inclusion dementia (MNDID) [23,57].

Non-FTLD pathologies have also been described in PPA patients such as Alzheimer's disease (AD) [12], Creutzfeldt-Jakob disease (CJD) and Lewy body disease (LBD) [5]. Similar to the imaging findings, left greater than right-sided pathological involvement is typical of PPA.

## 2. Symptomatology and diagnosis

### *2.1. Clinical presentation*

According to current research criteria for PPA, symptoms such as apathy, disinhibition, forgetfulness, visuospatial symptoms, or sensori-motor dysfunction should not be prominent during the first two years of the illness [38]. Acalculia or ideomotor apraxia are the only disturbances that can be present along with the language deficits in the early stages of the disease. Remarkably, in one patient, isolated progressive language symptoms were reported for 14 years prior to the onset of other major cognitive deficits [53].

Neuropsychological testing in PPA patients can be particularly difficult to interpret for clinicians who are not experienced with this condition. It should be noted that language impairments may prevent reliable assessments of non-linguistic abilities, since nearly all neuropsychological tests require verbal instruction, verbal responses and covert verbal reasoning. Furthermore, often it is difficult to compare the variants described in the literature because different language tests have been used.

Word finding deficits are the most common initial symptom in PPA. They can be attributed to either semantic/lexical deficits or underlying speech output problems. In order to overcome these difficulties, the patient often uses different linguistic strategies such as *simplification* (the use of a generic and more common word instead of a more precise but less common one) *and circumlocutions* (talking around an idea when the exact word or set of words cannot be retrieved). Often, verbal comprehension is preserved in the initial stages of PPA, but semantic memory deficits and difficulty comprehending specific types of sentences are apparent in different clinical variants when specific language tasks are performed.

Through the years, different terminology has been used to describe the heterogeneous clinical presentations of PPA and terms such as "nonfluent", "fluent", "PPA with agrammatism", "aphemic", "semantic dementia" (SD) and "logopenic aphasia" have been reported [40]. Based on current clinical criteria developed by Neary and colleagues, PNFA, (also referred to as "PPA with agrammatism") [40] is characterized by labored speech, agrammatism in production and/or comprehension, and, variable degrees of anomia, with relative sparing of single word comprehension. Sentence comprehension is impaired for the most difficult morphosyntactic constructions, such as negative passives and object negative clauses [21,44, 60,63]. Apraxia of speech (AOS), a motor speech disturbance characterized by impaired planning of the articulation of the words [64], is commonly found in this variant. Patients make inconsistent articulatory errors and prosody and fluency are typically disrupted [13]. In the early 1990s, an additional syndrome called progressive anarthria was described, in which patients presented primarily with articulatory errors, dysprosody and AOS [3]. Chapman and colleagues [6] have named a similar disorder "progressive isolated motor speech disorder", while Fukui and colleagues have called it "primary progressive apraxia" [11] and others "aphemic variant" [7,28]. Despite the variability of the nomenclature, all the authors emphasize AOS characteristics as a dominant symptom in these cases. Therefore, we have classified these patients as having classical PNFA [47]. Whether this presentation is a separate entity or one possible presentation of PNFA with mainly speech impairment is still a matter of debate.

In SD, also called "fluent" aphasia, spontaneous speech is fluent and grammatically correct, despite the loss of word and object meaning [22]. Semantic paraphasias (meaning the substitution of a more familiar exemplar within the same category: "dog" for "hyena") are common in this variant. In the initial stage, articulation, phonology, syntax and repetition remain intact [22,55]. As the disease progresses, language output can become "empty", poor in nouns, consisting primarily of vague fillers, such as "thing", and function words, such as "this" or "the". Spontaneous naming of objects is greatly impaired and performance does not usually improve when multiple choices are provided [13]. SD patients should show relative sparing of visual processing in the first two years from onset [40].

"Logopenic" language output, characterized by word-finding difficulties and decreased output with syntactically simple but correct output has also been described in PPA. Phonemic paraphasias (meaning sound substitutions within single words "tamp" instead of "lamp") are characteristic of this variant [28,63]. Recently, this clinical presentation has been suggested to be a distinct variant [13]. Sentence comprehension is severely impaired for all but the most simple morphosyntactic constructions and repetition is also below the normal limits. Naming is significantly impaired with spared recognition on multiple choices. Single-word comprehension and semantic memory are within normal limits. The core impairment in this variant is thought to be a short-term phonological memory deficit [13].

A consensus comprehensive classification of PPA has not yet been established. The use of different neuropsychological instruments and the fact that patients may present with different symptoms at different stages of the disease further complicates diagnosis. In Table 1, we summarize the clinical and language features of PPA variants described in the literature.

The heterogeneity of the clinical picture described in the literature is partially the result of the different assumptions and methodology applied to patient populations. Confounding factors include the variability in the use of specific language tests and the definition of fluency and agrammatism. In our view, the "pure progressive anarthria" should be considered together with PNFA, because often the two syndromes overlap and few cases remain purely anarthric with no evidence of language problems throughout the progression of the disease. The logopenic variant should include "PPA with conduction aphasia" [19]. Finally the "PPA with verbal comprehension deficits" should be considered as an early presentation of SD, when single word comprehension is significantly impaired. However, in the future, the heuristic value of these divisions will need to be determined validated with biological and neuropathological measures.

### 2.2. Associated symptoms

Behavioral problems are present to different degrees within the three variants. SD patients can show a wide variety of behavioral symptoms; the most frequent being depression, overeating or changes in food preferences, loss of insight, repetitive motor behaviors, alterations in social conduct and emotional blunting [52, 58]. PNFA patients are thought to develop behavioral symptoms similar to FTD later in the course of the disease, but usually remain polite and socially adept throughout the early stages of their illness. The logopenic variant has still not been neuropsychiatrically characterized, but these patients do not seem to show the severe behavioral disturbances that are common in the SD group.

On neurological examination, mild motor symptoms are common and usually localized in the right hand or the right side of the body, reflecting the focality of the left brain involvement [28,32]. PNFA cases showed more frequently diffuse motor slowing, reduced dexterity, and mild rigidity. Recently, a patient with PNFA who developed sudden-onset extrapyramidal symptoms with dystonia and alien hand has been described, providing further evidence that PNFA and cortico-basal syndrome can present in the same patient at different stages of the same disease [14]. In contrast, semantic dementia patients, apart for language impairment and agnosia, often have normal neurological examinations [13,37].

Limb apraxia is relatively common in PPA, being one of the two non-language symptoms that can be present early in the disease. In a small study, 14 patients had instrumental apraxia in imitation and/or with pantomiming [25]. This impaired gestural imitation is consistent with disruption of a left fronto-parietal network.

### 2.3. Pattern of language impairment and neuropathology

The pattern of language impairment does not easily allow the clinician to predict the etiology of underlying neuropathology. Indeed, many different neuropathology syndromes have been observed in PPA, including FTLD, CBD, PSP and AD. Clinical-pathological correlation is difficult in many cases because speech and language assessments were not sufficiently detailed to establish the presenting PPA variant [20,45]. Nonfluent aphasia (comprising isolated speech impairments) is often the first symptom of CBD, before the onset of extrapyramidal symptoms [15]. Dysarthria, buccofacial apraxia and AOS seem to be more common in cases of CBD [36,37,41,51], but other aphasia symptoms have also been reported. Attempts to link CBD pathology to a specific language pattern have, to date, been somewhat inconsistent. Nonfluent cases with AOS have also been associated with PSP pathology: tau inclusions were found in left fronto-temporal cortex (instead of

Table 1
Clinical and language characteristics of PPA variants

| | Spontaneous Speech | Single Word Comprehension | Repetition | Naming | Sentence Comprehension | Reading | Associated Symptoms |
|---|---|---|---|---|---|---|---|
| Progressive Non-Fluent Aphasia (Hodges et al. 1996, Neary et al. 1998, Mesulam 2001, Grossman 2002, Gorno-Tempini 2004) | decreased fluency, articulatory errors and apraxia of speech ± agrammatism | spared | agrammatic errors | normal/variable anomia, greater for verbs | impaired only for complex sentences | impaired for both regular and irregular words | buccofacial and limb apraxia, mild controlateral motor symptoms |
| Semantic Dementia (Hodges et al. 1992, Snowden et al. 1992) | fluent and grammatically correct, semantic paraphasias | impaired | spared | impaired, greater for nouns | spared | impaired for irregular | multimodal semantic memory impairment (faces, objects, sounds); behavioural symptoms |
| Logopenic PPA (Gorno-Tempini et al. 2004) | word finding problem, phonemic paraphasia | spared | Impaired | impaired, but increased performance with recognition | impaired, also for the simple sentences | spared for single word | acalculia, apraxia |
| Progressive Anarhtria/Progressive Isolated Motor Speech Disorder (Broussulle et al. 1996, Fukui et al. 1996, Chapman et al.1997) | articulatory errors, stuttering, dysarthria, dysprosody | spared | Normal/decreased for articulatory errors | spared | mild impairment? | spared? | buccofacial apraxia |
| Progressive Aphasia with verbal comprehension deficits (Mesulam 2001) | fluent, with frequent word-finding pauses | Decreased | preserved/impaired | impaired (category-specificty e.g. animate vs inanimate) | NA | NA | NA |

Table 2
Post-hoc analysis of neuropsychological test in the three variants

| | SD N= 14 Mean (sd) | PNFA N= 17 Mean (sd) | LPA N= 11 Mean (sd) | NC N= 10 Mean (sd) |
|---|---|---|---|---|
| *Demographic* | | | | |
| Age | 63.8 (7.1) | 67.3 (8.9) | 68.0 (9.8) | 66.4 (10.4) |
| Education | 16.8 (2.4) | 15.4 (2.3) | 17.2 (3.3) | 16.3 (2.7) |
| Gender (M/F) | 9/5 | 5/12 | 7/4 | 4/8 |
| *Neuropscychological measures* | | | | |
| MMSE | 23.8 (4.5)$^{a}$ | 25.2 (4.9) | 21.0 (6.0)$^{a}$ | 29.7 (0.5) |
| Digit backward | 4.5 (1.1) | 2.9 (1.7)$^{a,d}$ | 3.2 (1.2)$^{a}$ | 5.0 (0.1) |
| Praxis | 12.2 (1.8)$^{a}$ | 11.5 (3.6)$^{a}$ | 12.5 (1.8) | 14 (0) |
| Calculation | 4.4 (0.8) | 4.4 (1.2) | 2.5 (1.5)$^{a,b,c}$ | 4.5 (0.5) |
| *Speech and language production* | | | | |
| WAB speech fluency (10) | 9.1 (0.7)$^{a}$ | 6.1 (3.9)$^{a,d}$ | 8.2 (1.5)$^{a}$ | 10 (0) |
| Apraxia of speech rating (7 max deficit) | 0 (0) | 3.0 (2.3) | 1.5 (1.7) | NA |
| Dysarthria rating (7 max deficit) | 0 (0) | 1.4 (2.0) | 0 (0) | NA |
| WAB repetition (100) | 91.5 (8.4) | 79.2 (22.8)$^{a}$ | 79.5 (20.7)$^{a}$ | 99.5 (0.9) |
| Semantic fluency | 4.57 (3.10)$^{a,b}$ | 9.8 (4.9)$^{a}$ | 7.4 (3.9)$^{a}$ | 20.6 (4.5) |
| Phonetic fluency | 6.35 (4.08)$^{a}$ | 5.5 (4.7)$^{a}$ | 7.9 (5.9)$^{a}$ | 16.2 (5.1) |
| *Lexical retrieval and word comprehension* | | | | |
| WAB word recognition total (60) | 50.5 (9.9)$^{a,b,c}$ | 59.6 (1.0) | 58.5 (1.5) | 60 (0) |
| Single categories (6) | | | | |
| Real objects | 4.7 (1.4)$^{a,b,c}$ | 6 (0) | 6 (0) | 6 (0) |
| Drawn objects | 5.3 (1.5) | 5.9 (0.2) | 6 (0) | 6 (0) |
| Shapes | 3.84 (1.99)$^{a,b,c}$ | 5.88 (0.33) | 5.9 (0.3) | 6 (0) |
| Letters | 6 (0) | 5.4 (1.9) | 6 (0) | 6 (0) |
| Numbers | 6 (0) | 5.9 (0.2) | 6 (0) | 6 (0) |
| Colors | 5.7 (0.6) | 6 (0) | 6 (0) | 6 (0) |
| Furniture | 5.5 (1.2) | 6 (0) | 6 (0) | 6 (0) |
| Body parts | 4.9 (1.2)$^{a,b,c}$ | 6 (0) | 6 (0) | 6 (0) |
| Fingers | 4.3 (1.7)$^{a,b}$ | 5.8 (0.7)$^{a}$ | 5.4 (0.7) | 6 (0) |
| Left and right discrimination | 4.1 (1.9)$^{a,b,c,}$ | 5.82 (0.72) | 5.20 (1.47) | 6 (0) |
| BNT (15) | 4.7 (3.7)$^{a,b,c}$ | 12.5 (2.8) | 9.18 (3.0)$^{a}$ | 14.3 (1.2) |
| *Sentence comprehension* | | | | |
| WAB sequential command (80) | 74.5 (7.2) | 71.9 (8.7)$^{a}$ | 65.0 (18.9)$^{a}$ | 80 (0) |

$^{a}p < 0.05$ vs Control.
$^{b}p < 0.05$ vs NFPA.
$^{c}p < 0.05$ vs LPA.
$^{d}p < 0.05$ vs SD.

the “classical location” in precentral gyrus, subcortical areas and brainstem) [10,24]. The most common pathology in one study of 18 SD cases is MNDID (13 cases) [9], distantly followed by Pick’s disease (3) and AD (2).

AD is probably the second most common neuropathology associated with PPA, and occurs with both fluent and nonfluent predominant clinical syndromes [33,49].

Recently two series of pathologically confirmed PPA cases have been described. One showed that “possible PPA” (because of early memory impairment) had AD pathology; while “probable PPA” cases were associated with FTLD pathology (MNDID, CBD and Pick’s disease) [31]. The other [33] showed that nonfluent cases were more associated with tauopathies, while fluent cases showed predominantly MNDID; in both groups AD was the second most frequent diagnosis.

Future studies will need to include adequate samples to represent each PPA variant and a comprehensive language battery that can differentiate the variants during life. Finally, these batteries should be designed to detect patients with very mild impairments.

## 2.4. Neuroanatomy

Neuroimaging and neuropathology studies have shown left perisylvian involvement, affecting the language network, in most PPA patients [12,26,44]. SD patients, studied with Voxel Based Morphometry

(VBM), have significantly more atrophy in the medial and anterior temporal lobes than FTD [52] and AD [2]; these areas are involved in semantic memory. PET studies found left frontal hypometabolism in PNFA patients which might explain the agrammatism and AOS characteristics of these patients [17,46,61,62]. A recent study compared three PPA variants (PNFA, SD and LPA) in the same setting [13]. In addition to the areas previously reported, PNFA cases also showed atrophy in motor and premotor cortex. In our opinion, the involvement of motor cortex in PNFA provides further evidence that CBD could be the underlying pathology in some of these patients. Findings within the SD group showed atrophy in medial and lateral portions of the anterior temporal lobes bilaterally, confirming the results of previous studies. In the LPA group, atrophy was localized to the left inferior and medial parietal lobe, the posterior third of the middle temporal gyrus and the superior temporal sulcus and the left hippocampus. Atrophy in the LPA variant was more posterior than in the SD group, but the two variants overlapped in the middle and posterior thirds of the middle temporal gyrus. The temporo-parietal and hippocampal pattern of atrophy seen in LPA has previously been reported in AD patients [4,18], suggesting that AD pathology might be a common etiology in this variant.

Recent work compared a group of 11 PPA patients with 14 normal controls for studying the activation of the language network in this particular population (Sonty et al., 2003). They used phonological and semantic tasks with functional magnetic resonance imaging: PPA subjects showed overall increased activity in regions not typically activated by language tasks in normal subjects, such as left intraparietal sulcus, precentral gyrus and right fusiform gyrus. These results might be interpreted in two different ways: as compensatory neuronal strategies, with recruitment of areas outside the language network or defective inhibition, secondary to disintegration of specific neuronal pathways. In our view the first hypothesis is more plausible because subjects performed comparably to the controls, although with longer reaction times.

### 2.5. Hereditability

The hereditability of PPA is not well understood. Morris [42] described a family with four affected members with a clinical syndrome characterized by memory loss, language dysfunction, overeating and behavior and personality changes that he called Hereditary Dysphasic Disinhibition Dementia (HDDD1). Since then, few families have been described with progressive aphasia as the dominant phenotype [1,34,35,43,45]. The families described by Basun, and coauthors [1] had autosomal dominant inheritance. Linkage to chromosome 17q21-22 in HDDD type 2 has been found [35], as well as to chromosome 17q21 [1], yet, the genetic mutation causing these aphasic syndromes still remains to be determined. The clinical phenotype of the family described by Lendon had onset with memory and language deficits such as decreased and hesitant output and dysnomia. Behavioral symptoms often occurred earlier, while parkinsonian symptoms started at different points during the disease.

In sporadic cases, the homozygosis for the allele H1 of the tau genotype confers a higher risk for PPA [56]. These cases might share the same genetic vulnerability of sporadic PSP and CBD where the association with the same allele is strong. Another possible explanation of these findings is that some of the cases will go on to show CBD pathology, especially those with PNFA or those PNFA patients who begin with pure progressive anarthria.

The frequency of the allele $\varepsilon 4$ of the genotype ApoE, a risk factor for AD, is higher in the logopenic, posterior predominant cases [13]. In that study, the frequency of ApoE $\varepsilon 4$ was 20 percent in PNFA, 0 percent in SD, and 67 percent in logopenic cases. This genetic finding strengthens the hypothesis that logopenic PPA could often be the clinical presentation of asymmetric, focal AD pathology. Unfortunately, these last two studies discussed cases not pathologically confirmed and it is difficult to draw definitive conclusions.

### 2.6. Treatment approaches

A specific treatment for this patient population has not yet been discovered. Cholinesterase inhibitors, the drugs used in Alzheimer's disease, seem to be ineffective and in some cases worsen the behavioral symptoms. Selective serotonin reuptake inhibitors (SSRIs) and atypical neuroleptics can be used to control behavioral and mood symptoms, but no study has directly addressed their efficacy for improving language symptoms in this group of patients. Recently, a small double-blind, placebo-controlled cross over study, involving six PPA patients, tested bromocriptine for seven weeks: the dopamine agonist showed a mild slowing of language deterioration, but the benefits were limited [50].

## 3. Neuropsychological; language and neuroanatomical analysis of PPA

### 3.1. Cognitive results

We analyzed a sample of 42 consecutive PPA patients (17 PNFA, 14 SD and 11 LPA), (31 of them having already been described in a previous paper [13]) compared to 10 healthy elderly controls seen at the Memory and Aging Center at the University of California at San Francisco. All three PPA variants were determined based upon the criteria previously outlined. Our goal was to determine whether there were any distinguishing features related to demographic, neuropsychological or linguistic features of these three PPA subtypes.

Demographic variables were age, sex, education and disease duration. Neuropsychological variables were Mini Mental Status Examination (MMSE), digits backwards, phonemic and semantic fluency, praxis, calculation, 15 items from the Boston Naming Test, a spontaneous speech sample (fluency and information content), auditory word recognition, sequential commands and repetition subtests of the Western Aphasia Battery [27].

ANOVA or the Kruskal Wallis non-parametric test were used to determine overall group differences. Scheffe and Mann-Whithney tests were used to do post-hoc analysis on cognitive measures. These results are briefly described below.

Age, education and disease duration were not significantly different between patients and controls or within the three variants. As expected, results from neuropsychological testing showed significant differences when each PPA variant was compared to controls and to each other.

#### 3.1.1. Progressive nonfluent aphasia

Patients with PNFA presented with halting, effortful spontaneous speech, AOS (14 out of 17) and/or dysarthria (6 out 17). (The most common dysarthria type was mixed (upper and lower motor neuron, 3 cases), while one case of each of the following types was also noted: lower motor neuron, upper motor neuron and hyperkinetic dysarthria. In some cases, AOS was so severe that a dysarthria classification could not be made. Information content was also significantly decreased, but this was clearly at least partially due to speech production impairments. Repetition subtest scores were significantly decreased. Semantic and phonemic fluency scores together with comprehension for complex commands were significantly lower than controls. Single word comprehension evaluated with the WAB auditory word recognition was normal. Confrontation naming, often administered in the written rather than verbal modality, was relatively spared; additional improvement was noted when multiple choices were provided. MMSE scores were within normal limits. Performance on the digit backward task was significantly lower from the control and SD groups. On praxis, PNFA patients had the lowest scores, although not significantly different from the other two variants. Calculation was spared. Decreased fluency, speech impairment and working memory deficits were neuropsychological hallmarks of this group. These results suggest an involvement of left inferior frontal gyrus and anterior insula [13,46,60].

#### 3.1.2. Semantic dementia

SD patients showed nearly normal fluency but decreased information content, because of poor naming of low frequency objects in the description of the picnic scene picture from the WAB. None of the SD patients had AOS and/or dysarthria; sentence comprehension was spared. Semantic fluency was significantly impaired compared to controls and PNFA; phonemic fluency was also significantly decreased. SD patients had significant difficulty with recognition of objects, body parts, fingers and shapes; and they were not able to discriminate the left and right side of the body. Repetition and sequential commands did not differ from controls. Confrontation naming scores were lowest in the SD group and significantly different than PNFA, LPA and controls; multiple choices did not improve their performance. MMSE scores were impaired, while scores on the digit backward task were within normal limits and significantly better than in the other two variants. Praxis was significantly impaired, perhaps due to difficulty with object recognition (e.g., hammer, saw), as previously mentioned. Calculation was normal. This neuropsychological profile reflects a loss of knowledge regarding objects, actions and words. These results suggest an involvement of anteroinferior-medial temporal gyrus, which has been verified with neuroimaging [8,13,59] and pathologic studies [9]. Knowledge of action might be more impaired when the disease progressed to the frontal lobe [54], although the atrophy is only mild in this area [9].

#### 3.1.3. Logopenic progressive aphasia

LPA patients had spontaneous speech scores that fell between the other two variants. Speech output was slow and marked by word-finding difficulty. In some cases, phonemic paraphasias were difficult to differ-

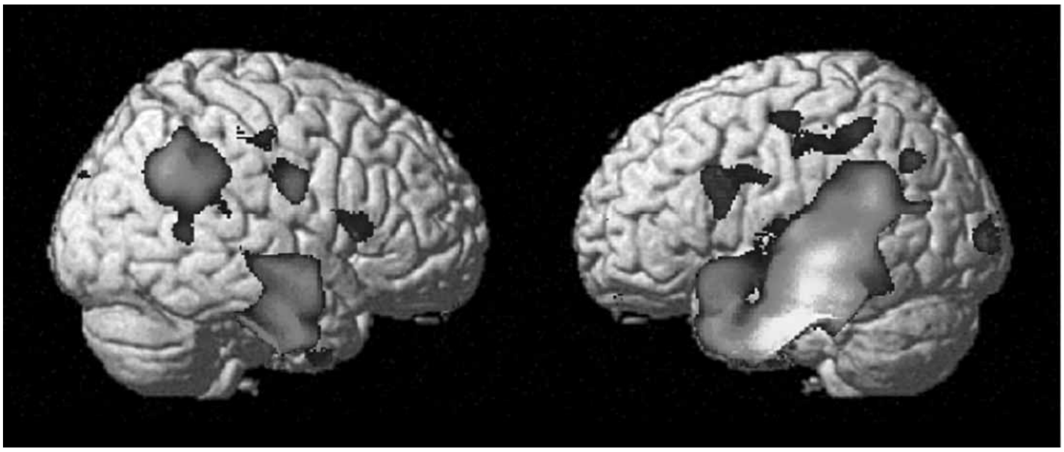

Fig. 1. Patients versus control. Involvement of a large network centered in the perysilvian region and left temporal lobe.

entiate from a "true" motor speech deficit, such as AOS. Phonemic and semantic fluency scores were significantly lower than controls, repetition scores were significantly decreased and the sequential commands subtest was more impaired than in the PNFA and SD groups. Single word comprehension was within normal limits. Confrontation naming test performance was impaired but, as opposed to SD, recognition on multiple choice partially improved the performance. Interestingly, this group was the only one in which calculation deficits were noted and MMSE scores were the lowest, although not significantly different than the other two variants. Scores on the digit backward task were also significantly impaired. Praxis scores were also significantly impaired. Overall, the LPA patients presented with prominent acalculia, and deficits in syntax comprehension and verbal rehearsal, suggesting left posterior temporal cortex and inferior parietal lobule atrophy [13].

In summary, SD patients showed the most consistent cognitive profile, easily distinguishable from the other two variants. PNFA and LPA performances overlapped in many cognitive tests. However, the mechanism behind the deficit was clearly different. For instance, while defective repetition was due to speech output difficulties and agrammatism in PNFA; phonological memory impairments probably accounted for poor repetition in LPA patients. Classic aphasia batteries such as the WAB, originally created for vascular aphasic patients, often fail to distinguish between variants of PPA.

### 3.2. Imaging results

We performed a structural MRI anatomical analysis of 51 MRI scans (39 images obtained from each patient described above and a group of 12 normal controls). Three patients were excluded from the analysis because they did not have an image acquired within six-months of the clinical and neuropsychological evaluation. MRI images were obtained on a 1.5T Magnetom VISION system (Siemens, Iselin, NJ). We analyzed the images with VBM, a technique for the detection of regional brain atrophy by voxel-wise comparison of gray matter volumes between groups of subjects. The technique includes an image preprocessing step (spatial normalization, segmentation, modulation, and smoothing) followed by statistical analysis. Grey matter volumes for each subject, age and gender were entered into the design matrix as nuisance variables. Regionally specific differences in gray matter volumes were assessed using the general linear model, and the significance of each effect was determined using the theory of Gaussian fields. Specific statistical analyses were performed to investigate the overall network of regions involved in PPA. We accepted a statistical threshold of $p < 0.05$ corrected for multiple comparisons for the whole brain, but we also report areas at $p < 0.001$ uncorrected if comprised in the left perysilvian language regions.

The results of the analysis (Fig. 1) showed that PPA diagnosis correlated with a large area in the left hemisphere that included inferior ($p < 0.05$, corrected), middle and superior temporal gyri ($p < 0.001$, uncorrected) together with left inferior frontal gyrus, ($p < 0.001$, uncorrected), insular ($p < 0.05$, corrected) and parietal inferior area ($p < 0.001$, uncorrected).

## 4. Conclusion

Isolated progressive speech and language difficulties are often the first symptom of left hemisphere focal forms of neurodegenerative diseases, particularly FTLD and CBD. AD patients also present with atypical focal cognitive manifestations, including fluent and nonfluent progressive aphasia. The variety of clinical presentations, anatomical areas involved and the multiple pathologies reflect the heterogeneity of the PPA population.

The clinical syndrome of PPA is associated with at least three distinct variants: PNFA, SD and LPA, but comprehensive language testing is often required to identify these anatomically, genetically and pathologically distinctive subtypes.

The heterogeneity of the clinical syndrome has relevance to neuropathology. We suspect that posterior variants of PPA will often show AD neuropathology, while frontally predominant cases will often show CBD at post-mortem. Language and neuroimaging testing help to separate these anatomical subgroups, although some patients will be difficult to classify into a single subtype.

Future studies should consider PPA variants as separate entities and use an integrated approach to collect longitudinal clinical, cognitive, and neuroimaging information as well as genetic and pathological data. Clinical, imaging and pathological information could then be correlated in order to better understand the pathogenesis and the specific etiology of the single variants. Since FTLD-like pathology might not be the underlying etiology in all variants, future studies investigating new treatments should also differentiate the three variants.

## Acknowledgement

The authors are supported by the following grants: 1 P50 AG-03-006-01 NIH/NIA – Alzheimer's Disease Research Centers, Core A and Core B; 03-75271 DHS/ADP/ARCC – (Dr. Miller); 5 P01 AG019724-02 FTD PPG –, Core A and Project 4. Wessinger Foundation (Dr. Miller); NINDS 1R01AG22985 and 04-35516 DHS/ADP (Dr. Gorno-Tempini).
